# Molecular Expression Profile Reveals Potential Biomarkers and Therapeutic Targets in Canine Endometrial Lesions

**DOI:** 10.1371/journal.pone.0133894

**Published:** 2015-07-29

**Authors:** Fabiana Azevedo Voorwald, Fabio Albuquerque Marchi, Rolando Andre Rios Villacis, Carlos Eduardo Fonseca Alves, Gilson Hélio Toniollo, Renee Laufer Amorim, Sandra Aparecida Drigo, Silvia Regina Rogatto

**Affiliations:** 1 Veterinary Clinic and Department of Surgery, São Paulo State University (UNESP), Jaboticabal, São Paulo, Brazil; 2 International Research Center (CIPE), A. C. Camargo Cancer Center, São Paulo, Brazil; 3 Veterinary Clinic Department, São Paulo State University (UNESP), Botucatu, São Paulo, Brazil; 4 Department of Urology, Faculty of Medicine, São Paulo State University (UNESP), Botucatu, São Paulo, Brazil; University of Bari, ITALY

## Abstract

Cystic endometrial hyperplasia (CEH), mucometra, and pyometra are common uterine diseases in intact dogs, with pyometra being a life threatening disease. This study aimed to determine the gene expression profile of these lesions and potential biomarkers for closed-cervix pyometra, the most severe condition. Total RNA was extracted from 69 fresh endometrium samples collected from 21 healthy female dogs during diestrus, 16 CEH, 15 mucometra and 17 pyometra (eight open and nine closed-cervixes). Global gene expression was detected using the Affymetrix Canine Gene 1.0 ST Array. Unsupervised analysis revealed two clusters, one mainly composed of diestrus and CEH samples and the other by 12/15 mucometra and all pyometra samples. When comparing pyometra with other groups, 189 differentially expressed genes were detected. *SLPI*, *PTGS2/COX2*, *MMP1*, *S100A8*, *S100A9* and *IL8* were among the top up-regulated genes detected in pyometra, further confirmed by external expression data. Notably, a particular molecular profile in pyometra from animals previously treated with exogenous progesterone compounds was observed in comparison with pyometra from untreated dogs as well as with other groups irrespective of exogenous hormone treatment status. In addition to *S100A8* and *S100A9* genes, overexpression of the inflammatory cytokines *IL1B*, *TNF* and *IL6* as well as *LTF* were detected in the pyometra from treated animals. Interestingly, closed pyometra was more frequently detected in treated dogs (64% *versus* 33%), with *IL1B*, *TNF*, *LBP* and *CXCL10* among the most relevant overexpressed genes. This molecular signature associated with potential biomarkers and therapeutic targets, such as *CXCL10* and *COX2*, should guide future clinical studies. Based on the gene expression profile we suggested that pyometra from progesterone treated dogs is a distinct molecular entity.

## Introduction

Cystic endometrial hyperplasia (CEH), mucometra, and pyometra are very common uterine diseases in countries where spaying healthy dogs is not a routine practice [1]. Among these diseases, pyometra is of particular importance in veterinary medicine due to its association with septicemia and toxemia [2]. Pyometra or chronic purulent endometritis affects approximately 25% of intact female dogs before the age of 10 and is characterized by bacterial infection and inflammation within the uterine cavity [3], [4]. In open cervix pyometra, inflammation leads to a distended uterus with a purulent vaginal discharge. Conversely, in closed pyometra an intrauterine purulent exudate accumulates leading to an increased risk of sepsis and subsequent death. Ovariohysterectomy is the most effective treatment in the prevention of overwhelming sepsis and disease recurrence [5].

Despite presenting distinct types of uterine fluid and degree of mucin hydration, mucometra and pyometra are considered similar diseases, both causing an accumulation of intrauterine fluid [6]. While pyometra presents an infected purulent fluid, mucometra is characterized by the presence of a sterile seromucous fluid within the uterine cavity [1]. Mucometra is thought to occur with CEH, leading to decreased fertility in breeding animals and having the potential risk of progression to pyometra [1], [2], [4].

The pathogenesis of pyometra is not completely understood. An association between pyometra and diestrus has been reported, confirmed by the absence of the development of pyometra in dogs who have undergone a bilateral oophorectomy [4]. CEH is the most common uterine disease in canines and has been proposed as a lesion that predisposes female dogs to pyometra [6]. CEH is a progressive proliferative process that is mediated by progesterone and potentially exacerbated by estrogen [2], [4]. In the pathogenesis of pyometra, progesterone has been reported by as being involved in endometrial gland secretion stimulation, suppression of the immune response and induction of cervical closure, providing a favorable environment for bacterial growth [2], [4]. In contrast, there is some evidence to suggest that uterine infection or endometrial irritation by foreign bodies may lead to an excessive endometrial hypertrophy and hyperplasia resulting in pronounced endometrial glandular proliferation. In addition, luminal epithelial cell secretion can initiate the development of pyometra or mucometra, depending on its origin, bacterial or not [2], [4], [7].

Although CEH, mucometra and pyometra are very common uterine diseases affecting intact female dogs, there have been a limited number of molecular studies performed on these disorders. To the best of our knowledge, only one study, using a limited number of cases, has reported the gene expression profile in pyometra samples in comparison with normal endometrium [8]. Thus, this study aimed to determine the gene expression profile of pyometra, comparing it with CEH and mucometra, as well as with endometrium samples obtained of healthy female dogs during diestrus. In addition, we sought to identify the molecular signature of closed pyometra, the most life-threatening form of the disease. These data have the potential to identify the biological mechanisms that contribute to uterine endometrial dysfunctions and reveal potential biomarkers that could be useful in clinical practice.

## Materials and Methods

### Animals

This study was performed according to the National and International Recommendations for the Care and Use of Animals. All procedures were performed under the approval of the Ethics Committee for Animal Experimentation from FCAV-UNESP (Permit Number: 008105/11). We obtained written owner consent before including any subject in the experiment. The animals were treated according to the norms of the Veterinary Hospital.

Endometrial samples were collected from female dogs, admitted to the Veterinary Hospital for elective or therapeutic ovariohysterectomy (OHE). The sample was composed of 16 cystic endometrial hyperplasia (CEH), 15 mucometra and 17 pyometra specimens (8 open and 9 closed-cervix pyometra samples). Two uterine samples with similar morphological features (approximately 1 cm^2^ each) per case were collected from mid-portion of the right and left horn for molecular and histological analyses, respectively. Luminal exudate was excluded from the endometrial samples. Normal endometrium, CEH, mucometra and pyometra cases were diagnosed based on clinical information, physical examination, radiographic and/or ultrasonographic images, and further histologically confirmed by two experienced pathologists (FAV and RLA). The determination of estrus cycle phase was performed through anamnesis, clinical control, vaginal cytology and serum progesterone levels [9,10]. Diestrus phase was also subdivided into early, mid- and late diestrus (S1 Table). Fifty-five animals were in diestrus and 14 in anestrus. Normal endometrial samples were obtained from 21 healthy female dogs during early diestrus. Seventeen of 69 dogs (1 from diestrus, 4 from CEH, 2 from mucometra and 10 from pyometra groups) were previously treated with exogenous progesterone-like compounds to suppress estrous signs. Clinical features of all animals are shown in Table 1. Statistical analysis included Chi-square or Fisher exact test to determine the association between the categorical variables.

**Table 1:** Clinical parameters in female dogs from diestrus, cystic endometrial hyperplasia (CEH), mucometra and pyometra groups.

Features	Diestrus	CEH	Mucometra	Pyometra
	N = 21 (%)	N = 21 (%)	N = 15 (%)	N = 17 (%)
**Age (years)**				
Median (range)	3 (1–9)	10 (3–16)	8 (3–13)	9 (6–14)
**Estrus cycle stage**				
Proestrus	-	-	-	-
Estrus	-	-	-	-
Diestrus	21 (100)	10 (62)	9 (60)	15 (88)
*Early diestrus*	21	9	3	9
*Mid-diestrus*	-	1	3	4
*Late diestrus*	-		3	2
Anestrus	-	6 (38)	6 (40)	2 (12)
**Breed**				
American Pit Bull Terrier	-	-	-	2 (12)
American Staff Terrier	-	1 (6)	-	-
Beagle	1 (5)	-	-	-
Boxer	-	1 (6)	1 (7)	-
Brazilian Mastiff	-	-	-	2 (12)
Brazilian Terrier	-	1 (6)	-	-
Cocker Spaniel	-	-	1 (7)	-
German Shepherd	-	2 (12.5)	1 (7)	-
Labrador Retriever	1 (5)	-	-	1 (5.5)
Pinscher	-	1 (6)	-	2 (12)
Poodle	-	2 (12.5)	-	2 (12)
Teckel	-	1 (6)	2 (13)	1 (5.5)
Mixed-breed	19 (90)	7 (44)	10 (67)	7 (41)
**Serum progesterone levels (P4) ng/mL**				
Mean ± SD error	20.13 ± 3.537	6.656± 2.034	3.576 ± 0.9891	9.893 ± 3.206
**WBC (x10** ^**3**^ **μL)**				
Mean ± SD error	7996 ± 1109	13070 ± 4327	9617 ± 1781	20789 ± 4535*
**HCT (%)**				
Mean ± SD error	40.38 ± 3.067	42.19 ± 4.657	37.58 ± 3.505	35.17 ± 2.994
**PLT (x10** ^**3**^ **μL)**				
Mean ± SD error	251.2 ± 19.350	357.3 ± 91.79	353.9 ± 64.3	232.7 ± 47.46
**Previous treatment with exogenous progesterone** **				
Yes	1 (5)	4 (25)	2 (13)	10 (59)
No	20 (95)	12 (75)	13 (87)	7 (41)

WBC, White Blood Count; HCT, Hematocrit; PLT, Platelet Count.

* Higher WBC was detected in pyometra compared with mucometra (*P* = 0.035), CEH (*P* = 0.0048) and diestrus (*P*<0.0001, Mann-Whitney test).

** Previous treatment with exogenous progesterone compounds to suppress estrous signs. A significant association of previous progesterone treatment and pyometra was detected (*P* = 0.001, Chi-square test).

### Blood Samples and Analysis

Before surgery, blood samples for hematological and progesterone (P4) serum levels were collected from the jugular vein using the vacuum system EDTA (BD Vacutainer Blood Collection Tube–BD, Franklin Lakes, New Jersey, USA) and EDTA-free (BD Vacutainer Serum Tube–BD, Franklin Lakes, New Jersey, EUA) respectively. Biochemical and hematological analysis were performed following routine protocol. Hematological parameters included hematocrit (HCT), white blood cell count (WBC) and platelet count (PLT). Serum P4 levels were measured by chemiluminescence immunoassay according to Tahir et al. [11]. Kruskal-Wallis or Mann-Whitney tests were applied to compare hematological data and P4 levels between the groups. Statistical analysis was carried out using SPSS version 17.0 (SPSS) and the GraphPad Prism 5 (GraphPad Software Inc.) software.

### Tissue samples and Histopathological Examination

Uterine tissues were collected immediately after OHE. Samples were both formalin-fixed for histopathology and snap-frozen in liquid nitrogen, the latter being immediately stored at -80°C for mRNA extraction. Formalin-fixed material was paraffin embedded, with hematoxylin and eosin (HE) slides prepared for histological diagnosis (CEH, mucometra or pyometra). Representative photomicrographs of endometrium tissue sections from diestrus, CEH, mucometra and pyometra groups are shown in S1 Fig.

### RNA extraction

Fresh frozen tissue samples were macrodissected using sterile scalpel blades, based on areas of endometrium identified following HE evaluation. Tissue samples were submitted to cleavage using lysing tubes in Precellys R tissue homogenizer equipment (BioAmerica Inc, Florida, USA). Total RNA was extracted using TRIzol reagent (Invitrogen Life Technologies Inc., Carlsbad, CA, USA) and the mRNA purified using the RNeasy MiniKit (Qiagen), according to the manufacturer’s recommendations. RNA samples were quantified on a Nano-Drop ND-8000 spectrophotometer (Thermo Scientific, Wilmington, NC, USA) and analyzed using Agilent 2100 Bioanalyzer 6000 Nanochip (Agilent Technologies Inc., Waldbronn, BW, Germany). Only samples with a RIN (RNA integrity number) higher than 7.0 were considered for use in the gene expression experiments.

### Gene expression profiling and pathway analysis

Gene expression profiling of 69 endometrial samples was performed using Affymetrix Canine Gene 1.0 ST Array (Affymetrix, Santa Clara, CA, USA). Data were extracted using the Affymetrix Genotyping Console (Affymetrix) and normalized using quantile normalization and robust multi-array analysis (RMA) background correction. Filtering characteristics of fold-change -2.0 to 2.0 and a FDR at P<0.05 were used to identify the differentially expressed genes. For normalization, processing and statistical analysis the limma v3.22.1 package was used (http://www.bioconductor.org/packages/release/bioc/html/limma.html). The Prism GraphPad software was used for Student’s t-test and Kruskal-Wallis test. The molecular processes, functions and molecular networks were further evaluated by analyzing differentially expressed genes using Ingenuity Pathways Analysis (IPA) (Ingenuity Systems, www.ingenuity.com). Protein-protein interaction (PPI) networks were annotated, visualized and analyzed using NAViGaTOR v2.03 (http://ophid.utoronto.ca/navigator/). Enrichment analysis by the IPA and PPI network analyses were performed based on human data. The microarray data are available on the Gene Expression Omnibus repository (GEO, http://www.ncbi.nlm.nih.gov/geo/), accession number GSE69481.

## Results

### Clinical data

Eleven different breeds, with the majority being mixed-breed dog, were represented in all groups (Table 1).

Higher serum levels of progesterone were observed in diestrus group compared with CEH, mucometra and pyometra (*P*<0.0001; *P*<0.0001; *P* = 0.006, respectively; S1 Fig and Table 1). Although pyometra samples showed a higher P4 levels in comparison with other uterine disorders, it was not statistically significant. Interestingly, significantly higher serum levels of P4 were detected in dogs with closed pyometra when compared with open pyometra (P = 0.0006, S2 Fig).

As expected, higher WBC were detected in pyometra group when compared with mucometra (P = 0.035), CEH (P = 0.0048) and diestrus (P<0.0001) (Table 1). No difference was observed for the other hematological parameters.

A significant association of previous exogenous progesterone treatment and pyometra (*P* = 0.001) was detected. The highest frequency of treated animals was observed in pyometra (59%, 10/17), followed by CEH (25%, 4/16), mucometra (13%, 2/15) and diestrus cases (5%, 1/21) (Table 1).

### Molecular analysis

**Fig 1:**
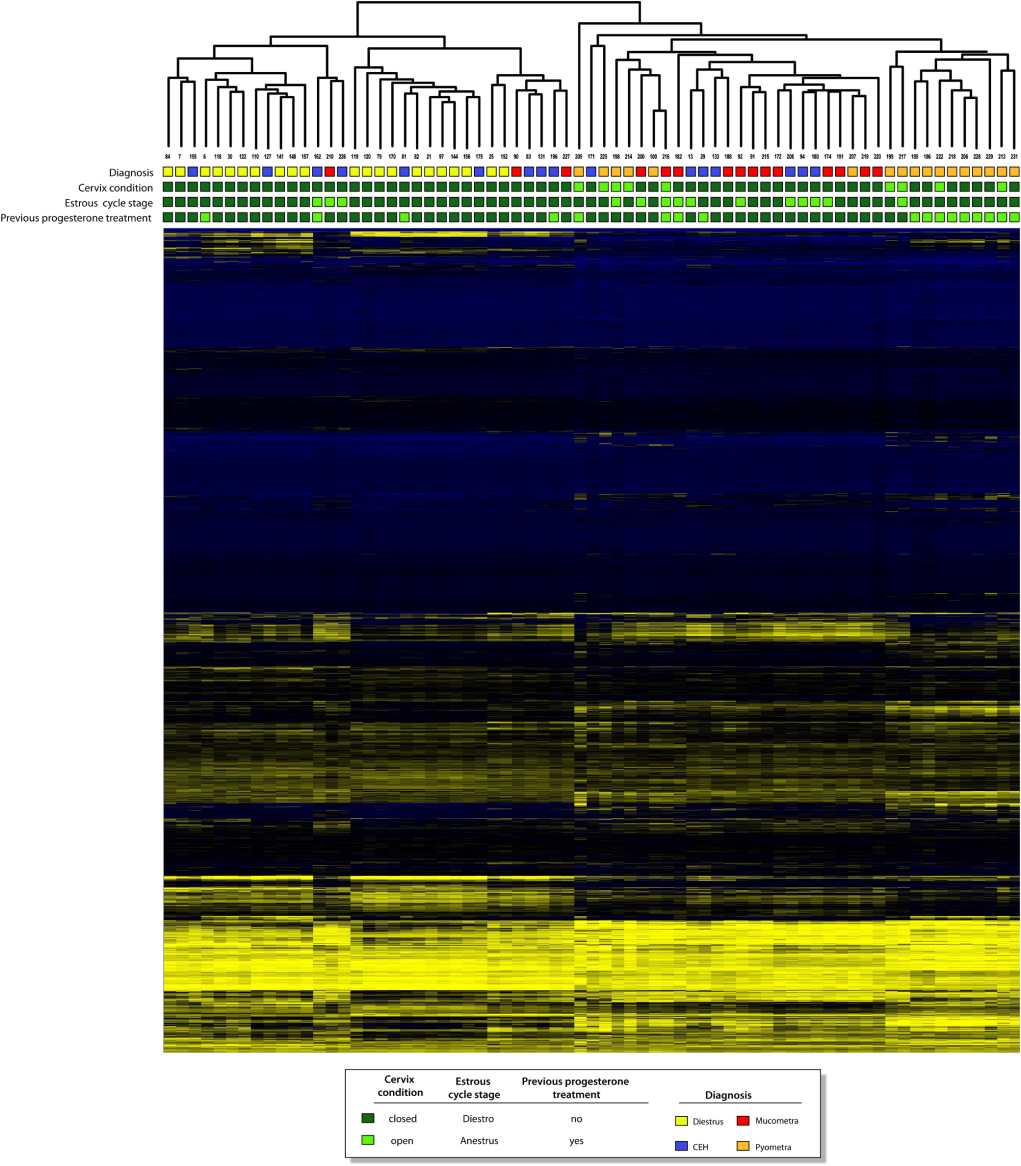
Hierarchical clustering analysis. Dendogram resulted from unsupervised analysis including diestrus, cystic endometrial hyperplasia (CEH), mucometra and pyometra samples. Cervix condition, estrus cycle phase and previous exogenous progesterone treatment status for each sample are shown.

Unsupervised hierarchical clustering analysis revealed two main clusters, with mucometra and pyometra samples showing a strong trend to cluster together, and diestrus and CEH samples mainly grouped in the other cluster (Fig 1). Pyometra showed 189 differentially expressed genes in comparison with other groups (diestrus, CEH and mucometra), with 169 overexpressed and 20 underexpressed (S2 Table). The *SLPI* (*secretory leukocyte peptidase inhibitor*) gene was detected as having the highest fold change, being 30 times more expressed in pyometra compared with other endometrial tissues (S2 Table). Among the highest upregulated genes in pyometra, three metalloproteinase genes (*MMP13*, *MMP1* and *MMP12*) and three S100 family members (*S100A12*, *S100A8* and *S100A9*) were observed. Conversely, the *EPHA7* (ephrin receptor A7) gene was detected as having the lowest fold change in pyometra.

**Fig 2:**
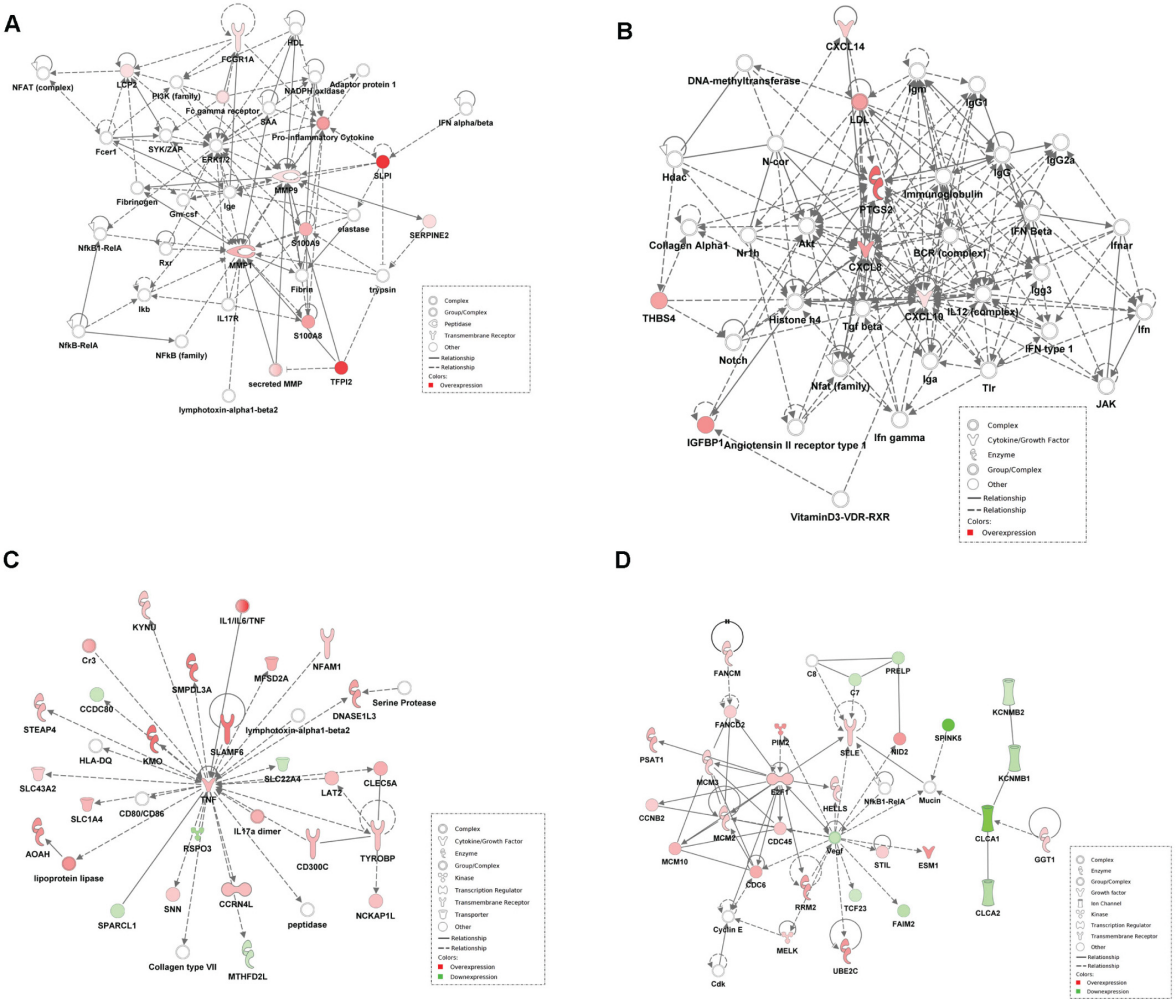
IPA network analysis. (A-D) Top networks identified with IPA software for differentially expressed genes in different group’s comparisons: A-B: Pyometra compared with diestrus, CEH and mucometra. A: Family members of the MMP and *S100* genes were detected as central nodes in pyometra and connected with pro-inflammatory cytokines. The *SLP1* gene (highest fold change in pyometra) is also depicted. B: Multiple interactions between the *CXCL8/IL8*, a pro-inflammatory gene, and other genes are shown, including the *PTGS2/COX2* gene. C-D: Pyometra of animals previously treated with progesterone compounds compared to pyometra of untreated dogs. C: The proinflammatory cytokine TNF was detected as central with multiple connections with different genes. D: *E2F1* (overexpression) and *VEGF* (underexpression) products in the treated group. The lines between genes represent known interactions, with solid lines and dashed lines representing direct and indirect interactions, respectively. Different node shapes represent the functional class of the gene product. Red and green nodes represent overexpressed and underexpressed genes in each comparison.

Canonical pathway and network analysis by IPA were performed for the differentially expressed genes in pyometra. Network analysis showed multiple interactions between the *MMP* genes and *S100* family genes detected with the highest fold change (Fig 2A). In addition, important interactions between overexpressed *CXCL8* gene, a chemokine that is one of the major mediators of the inflammatory response, and other genes were highlighted, including the *PTGS2/COX2* gene (Fig 2B).

A comparison between gene expression data in pyometra *versus* normal endometrium using the Hagman et al. (2009) [8] findings and our data (pyometra *vs*. diestrus) was also performed. Concordance of 58 differentially expressed genes was identified, with 33 being underexpressed and 25 overexpressed in pyometra (Table 2). The *SLPI* (*secretory leukocyte peptidase inhibitor*) gene was detected as being the top up-regulated gene in both studies. In addition, overexpression of the *MMP* and *S100* family genes was confirmed. To identify specific altered genes in pyometra, the list of differentially expressed genes detected in pyometra versus other groups (diestrus, CEH and mucometra) was compared with the list of 58 common genes detected by Hagman et al [8] data’s comparison. As a result, only 29 (50%) differentially expressed genes remained on the list, with 22 being overexpressed and 7 underexpressed (Table 2, highlighted in bold).

**Table 2:** Comparative analysis of the differentially expressed genes in pyometra *versus* normal endometrium as described by Hagman et al (2009) [8] and our data.

Genes	Fold change	
	(Pyometra *versus* Normal*)	(Pyometra *versus* Normal [8])
***SLPI***	50.26	344.8
***TFPI2***	47.10	48.1
***PTGS2***	36.59	88.7
***SMPDL3A***	27.78	194.4
*IGFBP1*	27.29	104.4
***IL8***	23.68	242.9
***S100A8***	23.02	56.6
*C6*	22.93	48.5
***THBS4***	22.82	31.2
***S100A9***	19.43	160.7
***SPP1***	18.54	27.7
***MMP1***	18.21	44.7
***SRGN***	12.96	53.2
***CXCL14***	12.74	47.0
***CCL2***	12.54	44.8
***SELL***	9.94	28.3
***MS4A7***	9.52	22.7
***SERPINE2***	8.33	25.6
***AOAH***	8.05	37.0
***MMP9***	7.31	29.4
***CASP4***	6.92	28.1
*CXCL10*	6.91	31.1
***FCGR1A***	6.67	42.5
***LCP2***	6.11	23.4
***C5AR1***	5.65	43.4
***EPHA7***	-22.05	-26.0
*SULT1D1*	-18.49	-30.6
*FEZ1*	-6.85	-10.7
*MSX2*	-6.15	-7.9
***LEF1***	-5.01	-7.4
*MSX1*	-4.99	-6.3
*TFCP2L1*	-4.89	-15.2
*ANK3*	-4.24	-8.1
*CMTM8*	-4.22	-5.7
*DEPDC7*	-4.09	-6.6
*RASGRP1*	-4.04	-5.4
*SLC30A2*	-3.99	-7.4
***RGS22***	-3.88	-8.9
*DLX5*	-3.79	-7.4
*GCLC*	-3.72	-7.0
***PPAPDC1A***	-3.68	-6.9
*CTH*	-3.58	-6.1
*RHPN2*	-3.40	-8.0
*ALDH1A1*	-3.19	-7.1
*TFF2*	-3.14	-9.2
*SNCAIP*	-3.08	-10.9
***EPHX2***	-2.98	-8.3
*GRIP1*	-2.96	-5.9
*WIF1*	-2.86	-5.5
*SH3BGRL2*	-2.85	-6.0
***EFHC2***	-2.72	-6.1
*PPP1R1B*	-2.65	-6.6
*HYI*	-2.55	-7.9
***ENPP6***	-2.40	-7.9
*FOXA2*	-2.38	-7.8
*STRBP*	-2.38	-6.2
*NAALAD2*	-2.29	-6.4
*NDP*	-2.26	-8.3

* Normal endometrium samples were obtained from dogs in early diestrus.

In bold are indicated those genes that were identified as differentially expressed when pyometra group was compared with the other groups (diestrus, CEH and mucometra).

The unsupervised clustering analysis (Fig 1) also revealed two distinct groups of pyometra, one of them being closer to diestrus and CEH samples, with the other clustering with CEH and mucometra samples, yet still isolated from them. Interestingly, the latter comprised a group of pyometra specimens obtained from bitches previously treated with exogenous progesterone compounds. In contrast, the pyometra samples that clustered near the diestrus and CEH samples were mostly obtained from untreated dogs (Fig 1). These findings indicated different molecular alterations in pyometra as a result of previous progesterone treatment. Estrous cycle phase and cervix conditions (open or closed) in each sample are also shown and were randomly distributed over the groups (Fig 1). In addition, the molecular profile was not correlated with histopathological features in each group (data not shown).

**Fig 3:**
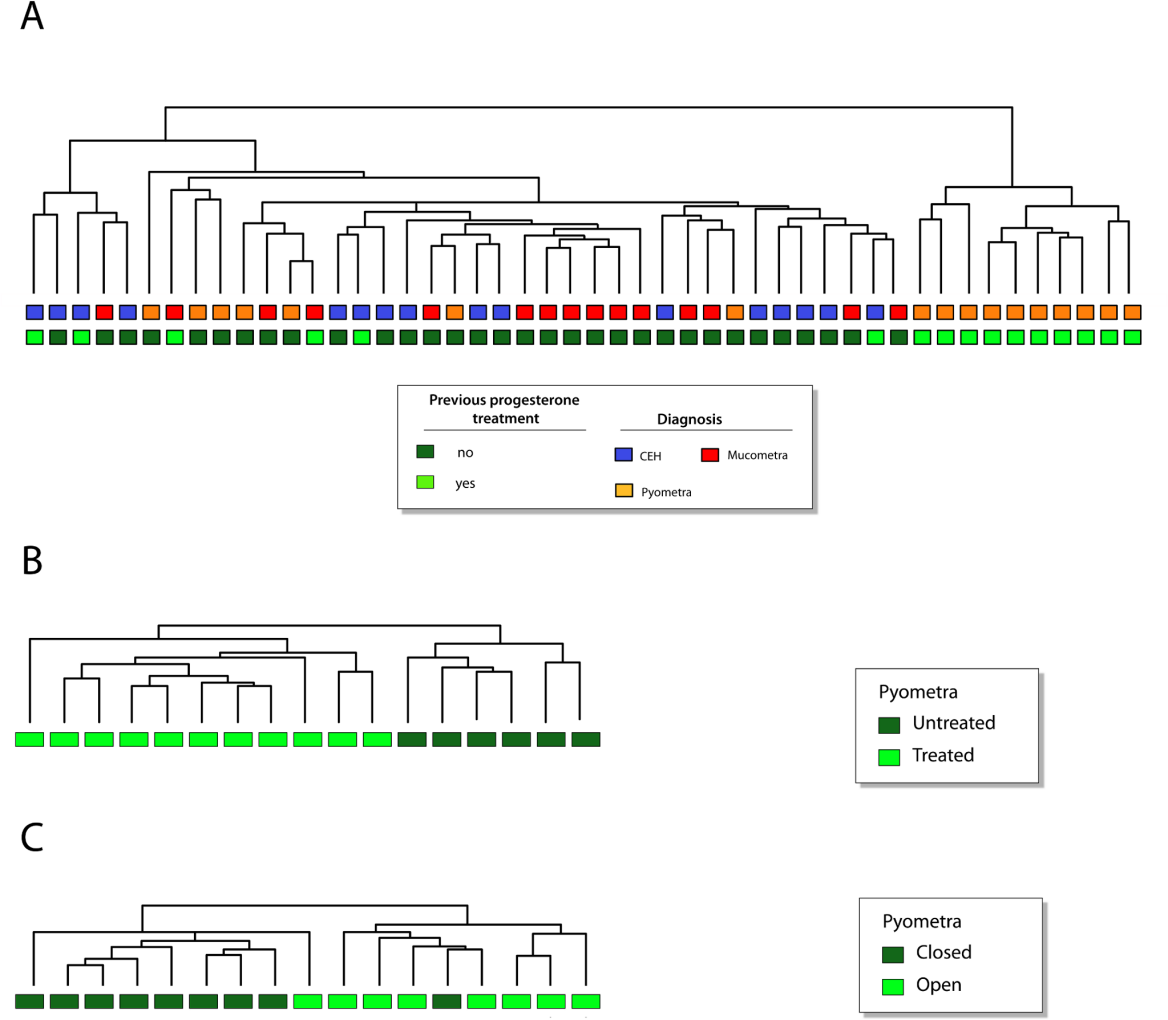
Supervised hierarchical clustering analysis of global gene expression data. Supervised analysis according to: **A:** absence or presence of previous exogenous progesterone treatment in female dogs with CEH, mucometra and pyometra; **B**. absence or presence of previous exogenous progesterone treatment in female dogs with pyometra. **C:** cervix condition in pyometra (open versus closed).

Further supervised clustering analysis was performed in CEH, mucometra and pyometra samples according to exogenous progesterone treatment status (Fig 3A). In accordance with previous unsupervised analysis results, two major clusters were observed, being one composed by all pyometra samples from treated dogs and the other cluster comprised by the pyometra samples from untreated animals as well as all by the other groups irrespective of treatment status (Fig 3A). These data suggest that pyometra from hormone-treated dogs present a distinct molecular signature. Therefore, the molecular profile of pyometra according to previous hormone treatment was investigated. Supervised clustering analysis revealed 194 differentially expressed genes, in treated (N = 10) versus untreated (N = 7) dogs, with 57 being underexpressed and 134 overexpressed (Fig 3B). The top 20 differentially expressed genes detected in this analysis are shown in Table 3. Interestingly, three members of the S100 family (*S100A8*, *S100A9* and *S100A12*) presented the highest fold changes among the up-regulated genes in pyometra from the hormone-treated group (Table 3). In addition, pro-inflammatory cytokines and chemokines (*TNF*, *IL1B*, *IL6*, and *CCL3*, among others) were also upregulated in the treated animals. Network analysis showed *TNF* as a central gene with multiple connections with other genes (Fig 2C) and an overexpression of the transcript factor *E2F1* gene interacting with different genes (Fig 2D).

**Table 3:** Top 20 differentially expressed genes in pyometra according to previous exogenous progesterone treatment.

Genes	Average treated group	Average untreated group	Fold change	*P* value	Adjusted *P* value
*S100A12*	10.76	7.18	11.98	0.0001	0.0028
*S100A9*	11.47	8.18	9.79	0.0000	0.0010
*IL1B*	7.89	4.64	9.46	0.0000	0.0010
*S100A8*	10.20	7.06	8.82	0.0000	0.0013
*PI3*	11.12	8.17	7.68	0.0001	0.0024
*EMR1*	9.29	6.52	6.83	0.0000	0.0022
*LTF*	11.40	8.68	6.59	0.0001	0.0036
*IL6*	8.05	5.38	6.36	0.0000	0.0016
*SLC7A11*	8.76	6.10	6.36	0.0000	0.0002
*CCNB1*	5.92	3.28	6.21	0.0001	0.0029
*PLEK*	9.79	7.20	6.02	0.0000	0.0010
*KMO*	7.81	5.25	5.91	0.0002	0.0044
*CLEC4E*	8.28	5.73	5.83	0.0000	0.0012
*DGAT2*	9.25	6.72	5.76	0.0000	0.0008
*CCL3*	9.54	7.02	5.74	0.0000	0.0011
*CHL1*	5.56	9.61	-16.57	0.0000	0.0001
*KRT5*	5.01	8.98	-15.67	0.0000	0.0009
*MAMDC2*	6.54	9.81	-9.64	0.0000	0.0000
*SPINK5*	4.48	7.62	-8.83	0.0004	0.0066
*ALDH1A1*	5.40	8.30	-7.47	0.0000	0.0012

* Differentially expressed genes were defined by a significant Bonferroni correction (*P*< 0.05).

**Fig 4:**
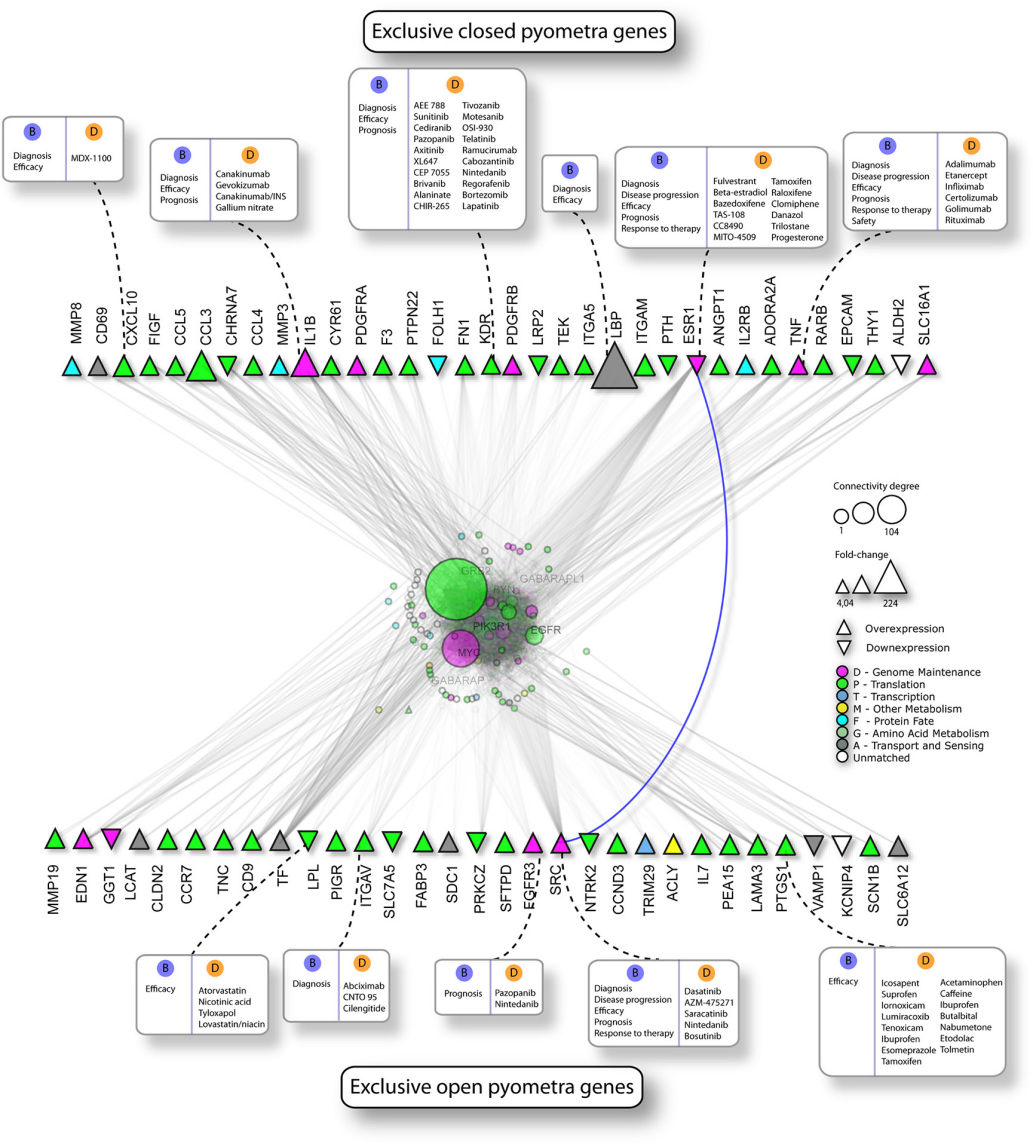
Protein-protein interaction (PPI) networks in closed and open pyometra. PPI networks based on altered genes exclusively detected in closed pyometra and open pyometra and their interactive partners built and visualized with Navigator v.2.3. Potential candidates for biomarkers (B blue circles) and targets for therapy (Drugs, D orange circles) in closed pyometra and open pyometra are highlighted. Triangles represent the genes with altered expression in each group and are color-coded according to Gene Ontology (GO). Upright and inverted triangles indicate overexpressed and underexpressed genes, respectively.

In order to identify a molecular signature for closed pyometra, a life-threatening condition, the expression profile of closed pyometra compared with open pyometra was investigated (Fig 3C). Eighty-two differentially expressed genes were detected, but no significant difference was observed after Bonferroni correction. Characterization of exclusive molecular alterations in closed and open pyometra was also sought, with the aim of identifying putative biomarkers. Firstly, two lists of significant genes exclusively expressed in open pyometra *versus* diestrus and closed pyometra *versus* diestrus were generated and further compared to reveal exclusively altered genes in each group. Interestingly, closed pyometra revealed 70 exclusively altered genes, while open pyometra had 34 (S3 and S4 Tables, respectively). The top five-upregulated genes in closed pyometra were *LBP*, *CCL3*, *IL1B*, *CXCL10* and *ITGAM*; while in open pyometra were *FABP3*, *IL7*, *TNC*, *SDC1* and *CLDN2*. With the aim of revealing potential biomarkers and therapeutic targets for closed pyometra, a gene set enrichment analysis by IPA was performed, revealing 21 genes exclusively expressed in closed pyometra (Table 4). The *IL1B* gene, which encodes a proinflammatory cytokine, was detected as the highest upregulated gene (Fold change = 9.29, Table 4). Overexpression of the *CXCL10*, *NNMT*, *MMP8*, *F3* and *TNF* genes was also identified in closed pyometra (Table 4). Thereafter, PPI networks were constructed using NAViGaTOR based on genes with altered expression detected exclusively in closed and open pyometra, in order to highlight potential biomarkers and/or therapeutic targets to therapy for each condition (Fig 4). In closed pyometra, four up-regulated genes were revealed as potential biomarkers and therapeutic targets, including *CXCL10*, *IL1B*, *KDR*, and *TNF*. The *LBP* gene, detected with the highest fold change, was indicated as a potential diagnostic marker in closed pyometra. In open pyometra, four overexpressed genes (*ITGAV*, *FGFR3*, *SRC* and *PTGS1*) were indicated as potential biomarkers and drug targets.

**Table 4:** Gene set enrichment analysis in closed pyometra detected by IPA analysis.

Gene	Location	Type(s)	Fold change
*IL1B*	Extracellular Space	Cytokine	9.29
*CXCL10*	Extracellular Space	Cytokine	6.22
*NNMT*	Cytoplasm	Enzyme	4.8
*MMP8*	Extracellular Space	Peptidase	3.83
*F3*	Plasma Membrane	Transmembrane receptor	3.37
*TNF*	Extracellular Space	Cytokine	3.07
*MMP3*	Extracellular Space	Peptidase	2.84
*KDR*	Plasma Membrane	Kinase	2.49
*RARB*	Nucleus	Ligand-dependent nuclear receptor	2.35
*PDGFRB*	Plasma Membrane	Kinase	2.34
*ANGPT1*	Extracellular Space	Growth factor	2.29
*ADORA2A*	Plasma Membrane	G-protein coupled receptor	2.27
*PDGFRA*	Plasma Membrane	Kinase	2.19
*IL2RB*	Plasma Membrane	Transmembrane receptor	2.1
*FN1*	Extracellular Space	Enzyme	2.1
*TEK*	Plasma Membrane	Kinase	2.07
*FOLH1*	Plasma Membrane	Peptidase	-2.02
*ALDH2*	Cytoplasm	Enzyme	-2.07
*ESR1*	Nucleus	Ligand-dependent nuclear receptor	-2.4
*EPCAM*	Plasma Membrane	Other	-2.36
*CHRNA7*	Plasma Membrane	Transmembrane receptor	-2.2

## Discussion

In this study, the global expression profile of pyometra was described in comparison to diestrus, CEH and mucometra, with a significant number of cases in each group. A distinct molecular profile for pyometra in female dogs previously treated with exogenous progesterone compounds compared with untreated dogs was described, indicating that they are, in fact, distinct molecular entities. This study also explored the molecular profile of closed pyometra, a condition that remains both a diagnostic and therapeutic challenge, in order to identify putative biomarkers and/or molecular therapeutic targets.

Unsupervised analysis of transcriptomic profiles allowed the identification of two major groups, with one of them having essentially diestrus and CEH samples, and the other composed of 7/16 CEH samples, 12/15 mucometra and all pyometra specimens. These data corroborate the current knowledge that CEH may predispose to the development of mucometra and pyometra. CEH can lead to endometrial thickening with subsequent accumulation of fluids within uterine cavity, increasing the risk of pyometra or mucometra development [1].

A cross study validation was performed via the comparison of our data (pyometra *versus* diestrus) with the Hagman’s data [8] revealing 58 genes in common in both studies, thus confirming our findings. However, 50% of these genes (29/58) remained as differentially expressed in pyometra after the comparison between pyometra versus other groups (diestrus, CEH and mucometra). This analysis, allowed the identification of altered genes exclusive to pyometra. The *SLPI* gene identified as the top overexpressed gene in pyometra encodes an antimicrobial peptide secreted by epithelial tissues. In addition, SPLI modulates infection and inflammation by neutralizing lipopolysaccharide (LPS) from Gram-negative bacteria impairing innate immune activation by toll-like receptors (TLRs) [12]. *SLPI* was previously reported as expressed in woman reproductive tract and induced by progesterone [13]. Up-regulation of the *SPLI* in pyometra may be endometrium-protective, both against microorganisms and from immune-mediated tissue damage.

Notably, both supervised analyses according to hormone treatment status evaluating all uterine disease samples (CEH, mucometra and pyometra) and only pyometra group confirmed a molecular signature for pyometra in treated dogs. These findings highlight the molecular alterations underlying the well-known association of pyometra with progesterone [2], [4]. Overexpression of *S100A8*, *S100A9* and *S100A12* genes were detected in pyometra, particularly in the hormone-treated animals. Several S100 proteins, including S100A8, S100A9 and S100A12, have been identified as endogenous danger-associated molecular patterns (DAMPs). DAMPs are intracellular molecules, which are released following cell death and can be recognized by the innate immune system, signaling tissue damage [14], [15]. S100A8 and S100A9 stimulate the production of the pro-inflammatory cytokines TNF, IL-6, IL-1B and IL-8 [14]. Accordingly, the *IL1B* and *IL8* genes were detected as overexpressed in pyometra group, being *IL1B*, *IL6* and *TNF* overexpressed particularly in pyometra of hormone-treated animals. Drugs targeting the S100A8/S100A9 complex leading to modulation of inflammatory response have been proposed in the treatment of cardiovascular disease [14]. Thus, high levels of *S100A8* and *S100A9* in pyometra may be a result of tissue damage leading to amplification and/or perpetuation of the local inflammation, being an attractive therapeutic target.

Overexpression of *PTGS2/COX2* was detected in pyometra, confirming previous report [16]. COX-2, also detected as differentially expressed in pyometra of hormone-treated dogs, is the more important source of prostaglandins and thromboxane A2 in inflammation. Furthermore, COX-2 is downstream of TLR signaling after activation by endogenous S100 proteins and other stimulus like LPS [15]. Thus, in pyometra a continuous activation of TLR by both endogenous and exogenous stimulus can lead to an exacerbated inflammatory response. COX-2 interacts directly with CXCL8/IL-8 and CXCL14 (Fig 2B), chemokines that were also overexpressed in pyometra. High serum levels of IL-8 were observed in dogs with pyometra, particularly in those that developed Systemic Inflammatory Response Syndrome (SIRS), suggesting that IL-8 may contribute to the development of a systemic disease in dogs with pyometra [17]. Overexpression of *CXCL14* can contribute to inflammatory cell infiltration in pyometra due to its chemoattractive action in immune cells, like monocytes and natural killer cells [18]. Both *IL8* and *CXCL14* are induced by *COX2*, thus selective COX-2 inhibitors could be an interesting treatment option in pyometra in terms of control the inflammatory response.

Overexpression of the *LTF* gene was detected in pyometra of hormone-treated dogs. Lactoferrin gene and protein overexpression were previously reported in pyometra [19]. Lactoferrin interacts with LPS on the bacterial surface and activates TLR-4 on the surface of phagocytes and epithelial cells. Interestingly, LTF can also be considered an inflammatory regulator by impairing LPS ligation to TLR and subsequent activation of the inflammatory response [20]. Thus, LTF might be induced in pyometra to counterbalance the inflammatory response. In contrast, the pro-inflammatory *TNF* gene was identified as a central gene in pyometra of hormone-treated dogs (Fig 3C). Considering that TNF has a pivotal role in inflammation, therapies targeting TNF could be considered in pyometra. In conclusion, a more pronounced inflammatory gene signature for pyometra of treated dogs was revealed, suggesting that repetitive progesterone exposure may contribute to bacterial colonization of the endometrium and an inflammatory response. This inflammatory environment enables the exposition and recognition of DAMPs by innate immune receptors, such as TLRs and production of pro-inflammatory cytokines and other inflammatory molecules, such as COX-2, aggravating the inflammatory process.

With regard to closed pyometra, the inflammatory process can progress to sepsis and SIRS, present either with or without infection [21]. Interestingly, a high frequency of closed pyometra was observed among hormone-treated dogs (7/10 cases compared to untreated dogs (2/7). Further studies are needed to confirm if repeated exogenous progesterone treatment predisposes to development of closed pyometra. Treatment with progesterone compounds to suppress heat cycles in female dogs is common in countries where spaying dogs is not a routine practice. Thus, these results are clinically relevant and neutering strategies in intact animals should be considered since treatment with progesterone compounds might ultimately produce undesired results. However, care should be taken considering that ovariohysterectomy during diestrus can lead to subtle progesterone decline and hyperprolactinemia with subsequent pseudopregnancy [22].

In this study, detection of exclusively altered genes in closed and open pyometra combined with enrichment analysis by IPA and PPI analysis were described, suggesting potential biomarkers and molecular targets for therapy. In closed pyometra, overexpression of *IL1B* and *TNF* were suggested as therapeutic targets, specifically anti-IL1 and anti-TNF antibodies. In addition, *CXCL10* overexpressed in closed pyometra is important in host immunity; but its excessive activation may lead to a detrimental immune response. The CXCL10 protein and its receptor (CXCR3) have been proposed as therapeutic targets in cancer and in immune-mediated diseases. Ongoing phase II clinical trials MDX-1100-anti-CXCL10, indicated here by PPI analysis in closed pyometra, have been conducted in inflammatory bowel disease and rheumatoid arthritis [23]. High serum levels of CXCL10, IL-6, IL-10, TNF and IL-8 were reported in a canine sepsis model [24]. Thus, CXCL10 in combination with other cytokines such as TNF are potential biomarkers for sepsis as a consequence of pyometra progression and also as targets in anti-inflammatory therapy in pyometra. *LBP* gene was also suggested as a diagnostic marker in closed pyometra. LBP is an acute-phase protein synthesized in the liver, but also by epithelial cells of other tissues, including the reproductive tract [25]. The LBP binds to LPS and other microorganism components leading to activation of the inflammatory response [25]. LBP serum levels have been proposed as a useful diagnostic marker in both urinary infections in children and atherosclerosis, as well as a prognostic marker in acute appendicitis [26]-[28]. Future studies are warranted to confirm LBP as meaningful diagnostic marker in closed pyometra. In open pyometra, *PTGS1/COX1* overexpression was detected as an exclusively altered gene. COX-1 is expressed constitutively in most cells and is also responsible for prostaglandin production during inflammation, which has the potential for being targeted by numerous anti-inflammatory drugs, including ibuprofen.

## Conclusions

To the best of our knowledge, this is the first report that described the expression profile of pyometra compared with other common uterine diseases, such as CEH and mucometra, in intact female dogs. In addition, a molecular signature for closed pyometra was described, which should be further explored to reveal prognostic and predictive biomarkers. Finally, distinct pyometra expression patterns from progesterone treated and untreated dogs were observed, with a more pronounced inflammatory signature in treated dogs, which suggests that previous progesterone exposure may contribute to an exacerbated inflammation in pyometra. A high frequency of closed pyometra in the hormone-treated group was observed and future studies are warranted to verify the impact of progesterone treatment on the development of closed pyometra.

## Supporting Information

S1 Fig Representative photomicrographs of endometrial specimens. HE stained endometrial sections obtained from diestrus, CEH, mucometra and pyometra groups (x5 and x10 magnification).
(TIF)

S2 Fig Serum levels of progesterone (P4) detected in diestrus, CEH, mucometra and pyometra (open and closed) groups. Mann-Whitney test.
(TIF)

S1 Table Diestrus subphases diagnostic criteria. (DOCX)

S2 Table Top 20 differentially expressed genes in pyometra samples compared to other groups (diestrus, CEH and mucometra samples). (DOCX)

S3 Table Exclusively altered genes in closed pyometra. (DOCX)

S4 Table Exclusively altered genes in open pyometra. (DOCX)
