# Supplementary figures and images for: Molecular Expression Profile Reveals Potential Biomarkers and Therapeutic Targets in Canine Endometrial Lesions
Source: PLoS One. 2015 Jul 29;10(7):e0133894. doi: 10.1371/journal.pone.0133894 (PMC4519320; doi:10.1371/journal.pone.0133894)

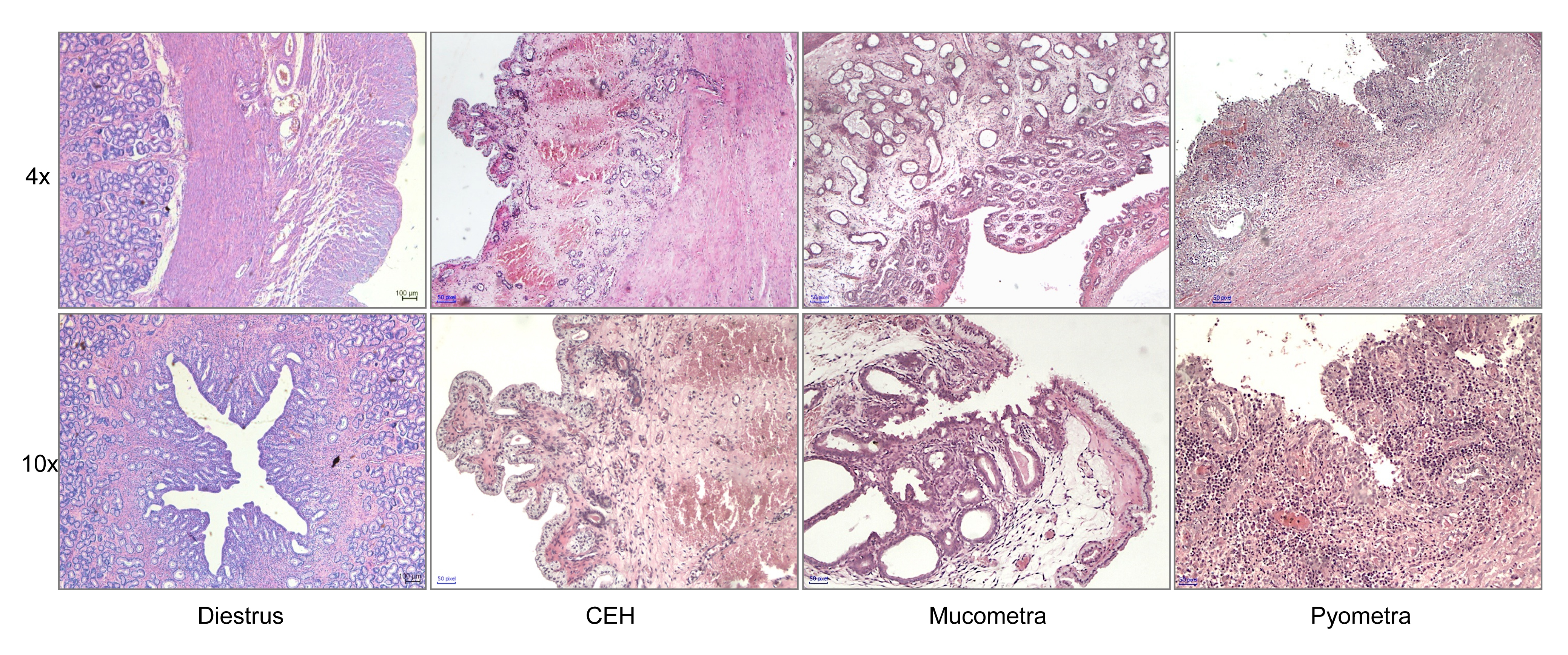

Supplement: S1 Fig — HE stained endometrial sections obtained from diestrus, CEH, mucometra and pyometra groups (x5 and x10 magnification). (TIF) [file pone.0133894.s001.tif]

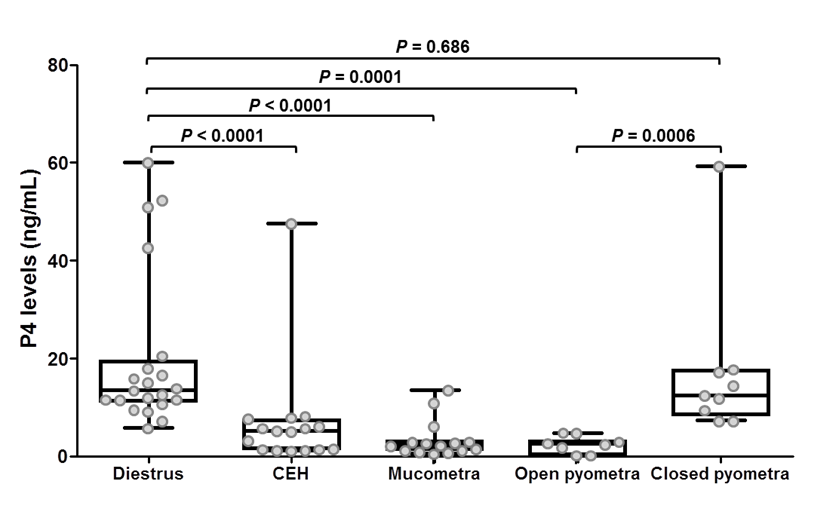

Supplement: S2 Fig — Mann-Whitney test. (TIF) [file pone.0133894.s002.tif]
