# Supplementary material for: Molecular Expression Profile Reveals Potential Biomarkers and Therapeutic Targets in Canine Endometrial Lesions
Source: PLoS One. 2015 Jul 29;10(7):e0133894. doi: 10.1371/journal.pone.0133894 (PMC4519320; doi:10.1371/journal.pone.0133894)
Supplement: S1 Table — (DOCX) [file pone.0133894.s003.docx]

| **Diestrus subphases** | **Vaginal cytology** | **P4 levels** | **Endometrial morphology** |
| --- | --- | --- | --- |
| Early diestrus | < 20% superficial cells. Presence of intermediate cells, neuthophils and small amount of basal cells | > 6ng/mL | Intense endometrial gland proliferation |
| Mid-diestrus | Mostly intermediate cells, few neutrophils and basal cells | 3 to 6 ng/mL | Moderate endometrial gland proliferation |
| Late diestrus | Most basal cells and some intermediate cells | < 1.7ng/mL | Lower endometrial gland proliferation |
