# Supplementary material for: Molecular Expression Profile Reveals Potential Biomarkers and Therapeutic Targets in Canine Endometrial Lesions
Source: PLoS One. 2015 Jul 29;10(7):e0133894. doi: 10.1371/journal.pone.0133894 (PMC4519320; doi:10.1371/journal.pone.0133894)
Supplement: S2 Table — (DOCX) [file pone.0133894.s004.docx]

| **Genes** | **Average**  **pyometra group** | **Average**  **other groups** | **Fold change** | ***P* value** | **Adjusted**  ***P* value*** |
| --- | --- | --- | --- | --- | --- |
| *SLPI* | 12.11 | 7.16 | 30.83 | 0.0000 | 0.0000 |
| *MMP13* | 10.08 | 5.57 | 22.81 | 0.0000 | 0.0000 |
| *MMP1* | 7.54 | 3.47 | 16.75 | 0.0000 | 0.0000 |
| *TFPI2* | 10.50 | 6.50 | 15.96 | 0.0000 | 0.0000 |
| *SMPDL3A* | 11.24 | 7.47 | 13.65 | 0.0000 | 0.0000 |
| *S100A12* | 9.50 | 5.80 | 13.04 | 0.0000 | 0.0000 |
| *S100A8* | 9.09 | 5.40 | 12.92 | 0.0000 | 0.0000 |
| *S100A9* | 10.31 | 6.85 | 11.04 | 0.0000 | 0.0000 |
| *IL8* | 10.63 | 7.18 | 10.91 | 0.0000 | 0.0000 |
| *MUC5AC* | 10.07 | 6.73 | 10.10 | 0.0000 | 0.0000 |
| *ACOXL* | 8.86 | 5.53 | 10.06 | 0.0000 | 0.0000 |
| *MMP12* | 8.36 | 5.14 | 9.36 | 0.0000 | 0.0000 |
| *IDO1* | 9.59 | 6.44 | 8.89 | 0.0000 | 0.0001 |
| *PI3* | 10.08 | 6.93 | 8.84 | 0.0000 | 0.0000 |
| *SBSN* | 9.99 | 6.92 | 8.39 | 0.0000 | 0.0012 |
| *EPHA7* | 6.90 | 9.58 | -6.42 | 0.0000 | 0.0179 |
| *PTGER3* | 6.97 | 9.23 | -4.80 | 0.0000 | 0.0000 |
| *ENPP6* | 7.35 | 9.28 | -3.80 | 0.0000 | 0.0016 |
| *LIN7A* | 5.41 | 7.31 | -3.73 | 0.0000 | 0.0001 |
| *ANGPTL1* | 5.23 | 7.01 | -3.43 | 0.0000 | 0.0010 |

* Differentially expressed genes were defined by a significant Bonferroni correction (*P*< 0.05).
