# Supplementary material for: Molecular Expression Profile Reveals Potential Biomarkers and Therapeutic Targets in Canine Endometrial Lesions
Source: PLoS One. 2015 Jul 29;10(7):e0133894. doi: 10.1371/journal.pone.0133894 (PMC4519320; doi:10.1371/journal.pone.0133894)
Supplement: S3 Table — (DOCX) [file pone.0133894.s005.docx]

| **Gene Symbol** | **Entrez Gene Name** | **Closed Pyometra**  **Fold change*** |
| --- | --- | --- |
| *LBP* | lipopolysaccharide binding protein | 14.98 |
| *CCL3* | chemokine (C-C motif) ligand 3 | 10.09 |
| *IL1B* | interleukin 1, beta | 9.29 |
| *CXCL10* | chemokine (C-X-C motif) ligand 10 | 6.22 |
| *ITGAM* | integrin, alpha M (complement component 3 receptor 3 subunit) | 4.97 |
| *DCDC2* | doublecortin domain containing 2 | 4.91 |
| *NNMT* | nicotinamide N-methyltransferase | 4.8 |
| *CCL4* | chemokine (C-C motif) ligand 4 | 4.58 |
| *CCL5* | chemokine (C-C motif) ligand 5 | 4.49 |
| *PTPN22* | protein tyrosine phosphatase, non-receptor type 22 (lymphoid) | 4.09 |
| *MMP8* | matrix metallopeptidase 8 (neutrophil collagenase) | 3.83 |
| *SLC16A1* | solute carrier family 16 (monocarboxylate transporter), member 1 | 3.47 |
| *F3* | coagulation factor III (thromboplastin, tissue factor) | 3.37 |
| *FIGF* | c-fos induced growth factor (vascular endothelial growth factor D) | 3.3 |
| *CD69* | CD69 molecule | 3.27 |
| *THY1* | Thy-1 cell surface antigen | 3.12 |
| *ITGA5* | integrin, alpha 5 (fibronectin receptor, alpha polypeptide) | 3.08 |
| *TNF* | tumor necrosis factor | 3.07 |
| *CYR61* | cysteine-rich, angiogenic inducer, 61 | 3.06 |
| *VWF* | von Willebrand factor | 2.98 |
| *MMP3* | matrix metallopeptidase 3 (stromelysin 1, progelatinase) | 2.84 |
| *SELE* | selectin E | 2.83 |
| *LCN2* | lipocalin 2 | 2.83 |
| *FSCN1* | fascin actin-bundling protein 1 | 2.68 |
| *CDH11* | cadherin 11, type 2, OB-cadherin (osteoblast) | 2.67 |
| *MEF2C* | myocyte enhancer factor 2C | 2.6 |
| *APOD* | apolipoprotein D | 2.54 |
| *PECAM1* | platelet/endothelial cell adhesion molecule 1 | 2.5 |
| *KDR* | kinase insert domain receptor (a type III receptor tyrosine kinase) | 2.49 |
| *TIMP4* | TIMP metallopeptidase inhibitor 4 | 2.46 |
| *GPR133* | G protein-coupled receptor 133 | 2.43 |
| *CSF3* | colony stimulating factor 3 (granulocyte) | 2.42 |
| *PTP4A3* | protein tyrosine phosphatase type IVA, member 3 | 2.38 |
| *CCL8* | chemokine (C-C motif) ligand 8 | 2.38 |
| *RARB* | retinoic acid receptor, beta | 2.35 |
| *PDGFRB* | platelet-derived growth factor receptor, beta polypeptide | 2.34 |
| *SNAI1* | snail family zinc finger 1 | 2.32 |
| *ANGPT1* | angiopoietin 1 | 2.29 |
| *ADORA2A* | adenosine A2a receptor | 2.27 |
| *SLC29A1* | solute carrier family 29 (equilibrative nucleoside transporter), member 1 | 2.26 |
| *CYTH1* | cytohesin 1 | 2.24 |
| *CDH5* | cadherin 5, type 2 (vascular endothelium) | 2.21 |
| *EMP3* | epithelial membrane protein 3 | 2.2 |
| *PDGFRA* | platelet-derived growth factor receptor, alpha polypeptide | 2.19 |
| *ABCG1* | ATP-binding cassette, sub-family G (WHITE), member 1 | 2.16 |
| *AKAP12* | A kinase (PRKA) anchor protein 12 | 2.14 |
| *KITLG* | KIT ligand | 2.13 |
| *IL2RB* | interleukin 2 receptor, beta | 2.1 |
| *FN1* | fibronectin 1 | 2.1 |
| *TEK* | TEK tyrosine kinase, endothelial | 2.07 |
| *NCF1* | neutrophil cytosolic factor 1 | 2.05 |
| *MME* | membrane metallo-endopeptidase | 2.02 |
| *ANPEP* | alanyl (membrane) aminopeptidase | 2.01 |
| *SNCG* | synuclein, gamma (breast cancer-specific protein 1) | 2.01 |
| *PTH* | parathyroid hormone | -3.15 |
| *LRP2* | low density lipoprotein receptor-related protein 2 | -3.04 |
| *INHBA* | inhibin, beta A | -2.91 |
| *WIF1* | WNT inhibitory factor 1 | -2.87 |
| *TSPAN8* | tetraspanin 8 | -2.7 |
| *KCNN3* | potassium intermediate/small conductance calcium-activated channel, subfamily N, member 3 | -2.42 |
| *ESR1* | estrogen receptor 1 | -2.4 |
| *EPCAM* | epithelial cell adhesion molecule | -2.36 |
| *CHRNA7* | cholinergic receptor, nicotinic, alpha 7 (neuronal) | -2.2 |
| *STYXL1* | serine/threonine/tyrosine interacting-like 1 | -2.15 |
| *GPX2* | glutathione peroxidase 2 (gastrointestinal) | -2.15 |
| *ISOC1* | isochorismatase domain containing 1 | -2.1 |
| *ALDH2* | aldehyde dehydrogenase 2 family (mitochondrial) | -2.07 |
| *PRDX1* | peroxiredoxin 1 | -2.07 |
| *ICA1* | islet cell autoantigen 1, 69kDa | -2.03 |
| *FOLH1* | folate hydrolase (prostate-specific membrane antigen) 1 | -2.02 |

* Fold change by comparing closed pyometra with diestrus.
