# Supplementary material for: Molecular Expression Profile Reveals Potential Biomarkers and Therapeutic Targets in Canine Endometrial Lesions
Source: PLoS One. 2015 Jul 29;10(7):e0133894. doi: 10.1371/journal.pone.0133894 (PMC4519320; doi:10.1371/journal.pone.0133894)
Supplement: S4 Table — (DOCX) [file pone.0133894.s006.docx]

| **Gene Symbol** | **Entrez Gene Name** | **Open Pyometra**  **Fold Change*** |
| --- | --- | --- |
| *FABP3* | fatty acid binding protein 3, muscle and heart (mammary-derived growth inhibitor) | 4.34 |
| *IL7* | interleukin 7 | 3.8 |
| *TNC* | tenascin C | 3.57 |
| *SDC1* | syndecan 1 | 3.48 |
| *CLDN2* | claudin 2 | 3.19 |
| *SFTPD* | surfactant protein D | 3.16 |
| *PIGR* | polymeric immunoglobulin receptor | 3 |
| *TRIM29* | tripartite motif containing 29 | 2.96 |
| *TMPRSS4* | transmembrane protease, serine 4 | 2.9 |
| *EDN1* | endothelin 1 | 2.79 |
| *SCN1B* | sodium channel, voltage-gated, type I, beta subunit | 2.67 |
| *PTGS1* | prostaglandin-endoperoxide synthase 1 (prostaglandin G/H synthase and cyclooxygenase) | 2.61 |
| *SLC6A12* | solute carrier family 6 (neurotransmitter transporter), member 12 | 2.43 |
| *MMP19* | matrix metallopeptidase 19 | 2.39 |
| *LCAT* | lecithin-cholesterol acyltransferase | 2.34 |
| *CD9* | CD9 molecule | 2.34 |
| *TF* | transferrin | 2.25 |
| *FGFR3* | fibroblast growth factor receptor 3 | 2.21 |
| *ITGAV* | integrin, alpha V | 2.18 |
| *CCND3* | cyclin D3 | 2.16 |
| *LAMA3* | laminin, alpha 3 | 2.13 |
| *CCR7* | chemokine (C-C motif) receptor 7 | 2.12 |
| *SRC* | SRC proto-oncogene, non-receptor tyrosine kinase | 2.09 |
| *PEA15* | phosphoprotein enriched in astrocytes 15 | 2.07 |
| *ACLY* | ATP citrate lyase | 2.04 |
| *GGT1* | gamma-glutamyltransferase 1 | -4.65 |
| *SLC7A5* | solute carrier family 7 (amino acid transporter light chain, L system), member 5 | -3.47 |
| *LPL* | lipoprotein lipase | -3.43 |
| *CD52* | CD52 molecule | -3 |
| *NTRK2* | neurotrophic tyrosine kinase, receptor, type 2 | -2.6 |
| *VAMP1* | vesicle-associated membrane protein 1 (synaptobrevin 1) | -2.3 |
| *KIAA1147* | KIAA1147 | -2.29 |
| *KCNIP4* | Kv channel interacting protein 4 | -2.17 |
| *PRKCZ* | protein kinase C, zeta | -2.05 |

* Fold change by comparing open pyometra with diestrus.
